# Socio-economic status, resilience, and vulnerability of households under COVID-19: Case of village-level data in Sichuan province

**DOI:** 10.1371/journal.pone.0249270

**Published:** 2021-04-29

**Authors:** Imran Ur Rahman, Deng Jian, Liu Junrong, Mohsin Shafi

**Affiliations:** 1 Center for Trans-Himalaya Studies, Leshan Normal University, Leshan, Sichuan, China; 2 School of Economics and Management, Leshan Normal University, Leshan, Sichuan, China; Neijiang Normal University, CHINA

## Abstract

This paper investigates economic impacts of COVID-19 on households based on differences in the socio-economic status (SES). We determine the household-level effects of the COVID-19 shock using income sources, types of industries, communities’ resilience, household susceptibility, and relevant policy measures. For this purpose, we used primary data of 555 households collected through snowball sampling technique using an online survey questionnaire from different villages mostly located in Sichuan Province, China. Using step-wise binary logistic regression analysis, we estimated and validated the model. Results suggest the use of SES as a better measure for understanding the impacts of COVID-19 on different households. We find that households with low SES tend to depend more on farmland income and transfer payments from the government. Contrarily, high SES households focus more on business and local employment as sources of income generation. Poor households were less resilient and more likely to fall back into poverty due to COVID-19, while the opposite stands true for non-poor households with high SES. Based on the estimations, policies encouraging employment and businesses complemented with loans on lower interest rates are recommended, which may increase the SES, thus minimizing vulnerability and enhancing the households’ resilience towards poverty alleviation and economic shocks.

## Introduction

Ever since the beginning of 2020, the world economy has been shocked by a new form of coronavirus termed as Novel Coronavirus Pneumonia (NCP) or COVID-19 [1]. COVID-19 has affected the socio-economic conditions throughout the world, especially in the case of developing economies and poverty. Poor households and households with low socio-economic conditions are suffering the most because of the pandemic. There seems a disparity in the impacts of COVID-19 at household levels due to differences in the socio-economic indicators of the households. Therefore, it is necessary to study the effects of external economic shocks on different households and the response of the households to such shocks. The economic factors may provide a better understanding of the differences in the factors and relevant policy implications for households.

The impacts of COVID-19 are diverse and spread across different sectors and regions. According to estimates of Sumner et al [2]. COVID-19 will impact the poor mostly in the regions of South Asia, Sub-Saharan Africa, and East Asia. Among Asian nations, China was also affected by the outbreak and faced various economic downturns in the first two quarters with lower production and growth rate. According to the national bureau of statistics of China, the income and consumption trends varied across rural and urban areas. Although the per capita disposable income of the rural households in the poverty-stricken areas showed a nominal increase of 2.7%, the real growth rate had decreased by 3.0% after measuring for price factors influence. In terms of the sources of income, including wages, salaries, and business, there is a slight increase in the nominal growth while net transfer income increases greatly by 9.2% due to the provision of the government. The net income from property declined by 0.5% in the first quarter as a result of the shock [3]. Table 1 shows the income trends.

**Table 1:** Income of rural residents in poor areas in the first quarter of 2020.

**Indicator**	**Income Level (Yuan)**	**Nominal Growth Rate (%)**
**Per Capita Disposable Income**	3218	2.7
**Income of Wages and Salaries**	1192	0.3
**Net Business Income**	1037	0.1
**Net Income from Property**	44	-0.5
**Net Income from Transfer**	945	9.2

Source 1: NBS China [3]

**Fig 1:**
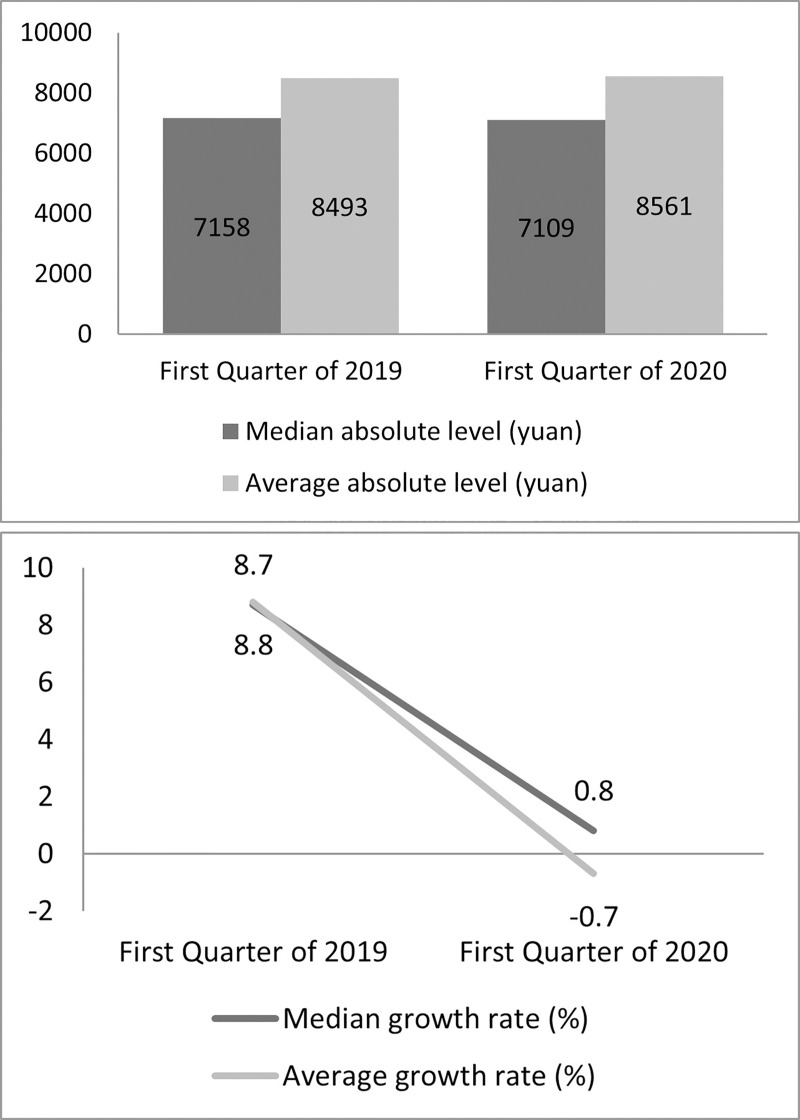
Quarterly changes in household income. (**A**) Median & Absolute Level of income; (**B**) Median and average growth rate of income. (Source 2 NBS China [4]).

Similarly, Fig 1A and 1B) shows the difference in the per capita disposable income of the Chinese residents in the first quarter of 2020 compared to the first quarter of 2019. The downward sloping lines clearly depict the decline in the median growth rates. Although there is a slight increase in the absolute and nominal value 8,561 Yuan, which shows an increase of 0.8% as compared to 2019; however, in terms of real value after adjusting for price factors, the income has declined by 3.9%, which can be attributed to the effects of COVID-19 on the households. For rural households, the actual decrease was 4.7%, which is higher than the urban areas due to the socio-economic status (SES) of the households in the rural areas.

The decline in the income in the case of the rural and urban areas also influenced the consumption patterns. The overall per consumption expenditure in nominal terms declined by 8.2% and in real terms by 12.5% in the first quarter. The rural and urban nominal and real per capita consumption expenditures also show downward trends. The real per capita consumption expenditure for rural households has been decreased by 10.7%; in the case of urban households it is decreased by 13.5% [4]. In the second quarter, the trends are expected to increase due to the re-opening of the main industries and the resumption of economic activities throughout China.

Although the spread of COVID-19 has impacted the majority of households in China, it is observed that some of the households are better-off during the pandemic as compared to the other households. Similarly, the poverty situation and socio-economic conditions of the households vary in outcomes and resilience towards external factors like COVID-19, with some poor and vulnerable households are assumed to have suffered more severely. These differences provide an opportunity for understanding why some households are affected insignificantly while others are affected significantly. Economic theories also provide various frameworks in estimating the effects of shocks on households based on income and consumption levels. Based on a similar assumption, the study of household’s socio-economic status (SES) for the estimation of the effect of COVID-19 on households may provide comprehensive and favorable outcomes in this case.

As various sectors of the economy are hit hard by COVID-19 but the poor and vulnerable communities are affected the most. Poor households and people living under poverty are considered to suffer due to the lockdown and closures of markets and business operations. Most of these people work on daily wages and may be unemployed with no alternate source of income. Governments across the world have stepped up relief and provision of subsidies to their citizens still the challenge for these vulnerable communities to recover hang in the air even after the pandemic is over [5]. In terms of developing countries, people living in low-income households face dual misfortunes: income-generating difficulties and vulnerability to socio-economics crises [6]. Poor households and people who are unprepared to cope with such shocks seem to be hit hardest by recession, inflation, disease and civil unrest. Similar trends are observed in terms of the impacts of COVID-19 on communities of different income groups. On contrary, rich households have greater access and a broader asset base to insurance and credit services, which will help cushion them against the effects of an external shock [7]. Thus, vulnerability and resilience of households to shocks or external factors can have diverse impacts based on the livelihood of the poor and non-poor communities. However, there is still a gap and dire need to address this issue and come up with a better and sustainable approach especially designed for protecting and supporting the poor and vulnerable communities. Based on these observations, it is also essential to understand and study the impacts of the COVID-19 on the different households and the main causes that highlight the disparities in the likelihood of the households to be in higher or lower SES level. However, there is still a gap and dire need to address this issue and come up with a better and sustainable approach especially designed for protecting and supporting the poor and vulnerable communities. Based on these observations, it is also important to understand and study the impacts of the COVID-19 on the different households and the main causes that highlight the disparities in the likelihood of the households to be in higher or lower SES level.

In this paper, we focus on the impact of COVID-19 on the household focusing on the SES differences in the villages of Sichuan Province of China. Our main objective is to determine the economics factors of households in response to COVID-19 based on SES differences. We also check the disparities in the level of resilience towards COVID-19 and households ability to counter falling under the absolute poverty by assessing the likelihood of the households SES conditions. The paper can contribute in providing better overview of the dynamics of the vulnerable households and suitable responses in fighting against external economic shocks. The outcomes may contribute in devising specific policies relevant to poor and vulnerable households.

The outline of the paper follows general rule. After introduction, the paper first summarizes the literature studies on impacts of pandemics and epidemics on different sectors and communities followed by vulnerability and resilience. Subsequently, the paper then provides the methodology adopted for estimations, followed by the main findings of the study and discussion on the output tables. Fnally the paper concludes and recommends policy implications.

## Literature review

The differences in the impact of a pandemic or an epidemic based on different aspects of the human life are evident from both historical and present-day perspectives. Similarly, in terms of COVID-19, the effects of the outbreak have shown variability across different regions and conditions of the world. At household level, the effects of the economic shocks have been adverse in terms of the economic status, resilience, and vulnerability of the households. At present due to the continuous spread of the virus, there are very few literature works that have focused on the impacts of COVID-19 shocks on poor household and poverty [8, 9]. Most of the poor live in developing nations and are more vulnerable to COVID-19 shocks and their effects on the status of the communities and households. Studies show 14–22 million people may fall below absolute poverty line due to 1% decline in global GDP [9]. Therefore, it is necessary to analyze the diverse impacts of COVID-19 on the status and livelihoods of different communities and households.

### Socio-economic status

Socio-economic status (SES) of a household is an important factor in signifying the outcomes related to shocks and vulnerabilities. It can affect the results of an economic phenomenon in different ways. According to the American Psychological Association, the Socio-economic status (SES) is a person or group’s social class or status which is often calculated by combining education, income and occupation [10]. As stated by Adler & Snibble [11] households with higher SES have better access to health care, housing, knowledge and nutrition as compared to lower SES households. Several research studies have focused on the impacts of different shocks on households based on the status. Some have highlighted the impacts of economic shocks such as joblessness or income decline and their effects on the household’s status [12, 13]. While others have argued on the health shocks effects on individuals health and in turn the productivity of the workers finally leading to decline in income level of the households [14]. There is still very few literature articles related to the impacts of shocks on the socio-economic status of different households and communities in the developing world.

Some researchers have highlighted the relationship between household status and shocks [15]. Other researchers argue that the effects of economic shocks on households are greater in households that are poor due to insufficient income to cushion against the external shock [16]. In terms of effects on communities and household, shocks at village level are usually not considered to affect the permanent income of the individual households. Others argued that household consumption declines due to negative shocks that impact the permanent income of the households [17, 18].

Economists have formalized different form of models for the estimation of household response towards risks and shocks. Consumption response to income shock is one such model which focuses on use of combination of consumption, income or wealth in response to shocks [19, 20]. Kruger and Perii [21] used consumption and wealth as household response towards the income shock. Nevertheless, the issue of which model fits the household response remains unclear. For this reason, this paper attempts to use the SES for the estimation purposes as it present a better proxy and a good indicator of the household’s economic situation.

### Vulnerability and resilience

The concepts of vulnerability and resilience are found in various disciplines and subjects. A body of literature has emerged in the past few decades that have focused on different aspects of vulnerability. The notion of vulnerability has become an influential theoretical method for defining the condition of susceptibility to risk, inadequacy, and marginality of both physical and social institutions, and for influencing normative study of behavior to increase quality of life through risk mitigation [22]. The term vulnerability, according to the World Bank [23], is “the likelihood that a shock will result in a decline in well-being”, while some researcher have described vulnerability as a state of defenselessness and exposure to shocks [24]. This definition can be further explained that any external shock that can lead to the decline of the livelihood of households including income and other socio-economic factors, determines the vulnerability of the households.

Some studies focused on presenting evidence of vulnerability by examining the methods used by rural households in low-income countries to deal with HIV/AIDS [25]. A study by Ndirangu explores how farm households in rural Kenya react to shocks and the impact on quality of life of household risk management strategies. The research explores two primary causes of rural vulnerability: the weather and AIDS. The study shows strategies for coping with shock includes dependence on collaborative opportunities such as casual loans and gifts [6]. The impact of vulnerability in our case can be assessed and analyzed empirically through the addition of vulnerability of the households against COVID-19 in the model. We check the vulnerability and attributes of it relevant to poor households. By doing, useful understanding and insights can be obtained that can enable policy makers in formulating better policies to avoid the risk related to poor households in case of shock.

Similarly, the concept of resilience has gained much attention in recent years especially in terms of poor households [26]. Resilience refers to the ability of households to cope with basic necessities of livelihood in case of emergency or shock [27, 28]. Resilience has been implemented by different scholars in diverse perspectives [29–31]. There is a broad consensus that resilience is not stagnant, but that it continues to evolve [29, 32], that needs further analysis and examination.

Previous studies have focused on the resilience of household in case of natural disasters while others have focused on resilience of households to the socio-economic shocks [33–35]. Resilience of households has also been studied in terms of ecological and environmental effects [36, 37]. Although different qualitative approaches have been undertaken in terms of analyzing the resilience but the studies based on quantitative resilience are scarce and limited [38, 39]. The resilience of households taking into consideration the livelihood of the households is also an important factor. Several studies have focused on this component in terms of external shocks [40, 41]. However, studies have focused on different aspects and factors linking resilience to household income and assets, yet there is a dire need to better understand the connection between these components in terms of rural households. This study will attempt to fill this gap and contribute to the empirical aspect of the literature.

In terms of measuring vulnerability and resilience, economists have argued on various economic factors including household income, status, capital, and access to social security nets [42–44]. In terms of understanding the response of household towards a certain external shock like the COVID-19 pandemic, resilience and vulnerability can be good measures. The impacts of COVID-19 on households are unprecedented and the responses are observed to vary from household to household due to the differences in the SES of the households. Similar, observations are reported in case of China. The assumption can be drawn that households with higher SES may have better coping strategy and resilience as compared to household with lower SES. In this paper, we analyze the difference in the households resilience based on SES using the survey data.

For empirical and quantitative analysis, some authors measured vulnerability based on its impacts on indicators of welfare of the households [45, 46]. Others estimated vulnerability in relationship to the poverty dynamics of the households. In this paper, we follow the approach of Vulnerability as Expected Poverty (VEP). The VEP approach is defined as the probability that the expected Income expenditure of a household will fall into poverty in the future [47]. This approach was also adapted in terms of poor households in developing countries by other scholars [7, 48, 49]. In case of previous studies, the vulnerability was estimated in case of poverty status of the households, which stood above the absolute poverty line. These measures did not consider the effects in case of households below the poverty level, which may provide a deeper understanding, and vulnerability effects on poverty status of households. Chaudhuri [47] pointed out that welfare status of the households tend to decline where the households poverty status is below poverty line as compared to households above poverty line due to the increase in vulnerability and risk. Based on this notion, we build our model and apply it in case of household status both above and below the poverty line, which will provide a better, and in-depth measure of the vulnerability of different households as compared to previous ones.

Additionally, some past literatures have also focused on different coping strategies across multiple domains including access to credit, government support, and help from family and friends, use of savings, and other such strategies [50, 51]. While, the coping strategies related to the SES of household especially poor households, have not been clearly observed [16]. Coping strategies towards shocks may differ and their impacts may vary based on the status of the households. In this paper, we observe a coping strategy specifically based on the significance of the policy for both poor and non-poor household status, which in this case are the loans provision on lower interest for business and its impacts on different households. There are very few research on the poor households susceptibility towards pandemic [52], there is still more to explore in terms of COVID-19 and its impacts on poor households and the response of the communities.

This paper attempts to provide the impacts of COVID-19 on household based on the SES of the households. Additionally, the paper further identifies the differences in the impacts of the outbreak taking into consideration the SES of the households by dividing the households into two groups (poor and non-poor households). Based on these differences, the resilience of the households towards a shock like COVID19 and the vulnerability of the households in face of coping with the outbreak are also analyzed and estimated for a deeper understanding of the differentiated effects of COVID19.

## Methodology

### Sample and data collection

For the empirical estimations and predictions of our model, we use both primary and secondary data from different sources and methods. For the collection of primary data, an online survey was developed. The online questionnaire was shared using different social media platforms, which made it easier for data collection. The data was collected from village representatives (potential key respondents). The questionnaire was filled by different households with majority from the villages of Sichuan province (475 in Sichuan while 80 from other provinces). A total of 555 questionnaires were completely filled through the online channels over a period of two weeks. The province includes least, middle, and highly developed regions similar to mainland China. Due to measures taken by the Chinese government to contain the spread of the virus, including social-distancing and travel restrictions, the data was collected through administering an online questionnaire, which is also consistent with previous studies [53, 54]. In case of Sichuan Province, there are about 183 counties that are different in terms of geo-political and socio-economic indicators [55]. For secondary data, online official websites were utilized. Health data and reports were collected from WHO website [56, 57], while data on Chinese villages was collected from National Bureau of Statistics China website [58].

The data was collected through the snowball sampling technique due to its time and resources-saving advantages, as argued by various researchers [59, 60]. The data was collected during mid of April 2020 for two weeks; at that time COVID-19 pandemic had already affected a large number of local people in terms of socio-economic and health effects of COVID-19.

We sought permission from the relevant community leader of every village prior to administering the questionnaire. Besides, the consent of each village representative was obtained prior to partaking in the survey. Each respondent was allowed not to answer any question accordingly. The current research was approved by the Ethics Committee of Leshan Normal University. Prior to taking part in the online questionnaire, informed consent was obtained from each participant. The purpose and content of the study were first introduced to the potential participants before partaking in the survey. After assuring respondents of complete anonymity, confidentiality, and other ethical considerations, they filled the online questionnaire. There was no electronic record of the participant’s consent, but all respondents agreed on the purpose of the research and took part in the online survey voluntarily.

### Measures

A questionnaire was developed to study and investigate the economic impacts of COVID-19 on households based on differences in the socio-economic status (SES). In this study we attempt to determine the household-level effects of the COVID-19 shock using different socio-economic factors. For this purpose, we asked several questions from participants such as SES level, household level (poor or non-poor), number of community groups in the village, per-capita annual disposable income (in normal times), main sources of income, industry involvement, agricultural supply level, cost of livestock breading (after COVID-19 outbreak), impact of COVID-19 on rural households to fall back into poverty, vulnerability, and resilience level of households, among others. For more details about the survey questions, see S1 File.

In case of the measurement some researchers argue that although the consumption patterns and disposable income are currently applied for the evaluation of the welfare status and living standards of the households [61] but these are not the only proxy variables for representation of welfare and status of the households. The variables like welfare and living standards are somewhat hard to observe directly. Instead, different proxy variables are utilized for the indirect measurement of such values. Therefore, it is possible to estimate new proxy variables as suggested by Massari [61] which can best define the changes and effects on households. Using the Bank of Italy SHIW data, he included a new proxy variable “real assets” in addition to disposable income and consumption expenditure. Similarly, in this paper we assume the SES of the household may provide a better measure as a proxy representing the welfare of the households.

We develop a simple model based on step-wise binary logistic regression in order to estimate our variables. As we used an online survey (questionnaire) for the collection of the data, so some variables in our data are coded as binary variables while others in categorical form. Based on the design of the questionnaires, the responses and the data, it is convenient to use the logistic regression approach for better estimates, good model-fit and unbiased estimates [62, 63].

### The model

The model predicts the impact of different sources of income, industries, and policies under COVID-19 based on the SES in the villages of Sichuan province, China. As mentioned earlier, the households are divided into poor/low SES and non-poor/high SES household based on the survey data. The household level SES is based on village representative’s identification of the average SES level of the households in the villages (high SES or low SES village) and the reply of the respondent’s in the questionnaire. Based on this fact, we assume SES to be a better indicator for the indirect impacts of COVID-19 in case of different SES levels of the households.

We construct our model using SES as a proxy for the household’s main indicators of income, consumption, assets and social indicators based on the national statistics of China. SES contributes and expands the multiple indicator [61, 64], by inclusion of social indicators such as education, members etc. with broader perspectives of the households status. As our dependent variable assumes binary values of 0 or 1, the binary logistic regression is adopted. In the literature works [62, 63], a simple logistic model equation is given by:

$$Y_{i}=\beta_{0}+\beta_{1}X_{i}$$ (1)

Where, *Y*_*i*_ is the linear function and the dependent variable, *X*_*i*_ denotes the independent regressors and β measures the coefficients of the regressors in the model.

Eq 2 can also be written in the form of,

$${Logit(Y}_{i})=\beta_{0}+\beta_{1}X_{i}$$ (2)

This represents a simple logistic linear regression equation for estimating binary. As the dependent variable in our model consists of binary values (0,1), it is better to treat the model as a linear equation [62]. As our dependent variable to be estimated is also binary coded, we can construct a binary logistic model, which is depicted as follows:

$${\mathrm{Logit}\;SES}_{i}=\alpha_{0}+\beta_{i}X_{i}+\gamma_{i}Z_{i}+\delta_{i}N_{i}+\mu_{i}$$ (3)

Where, *Y*_*i*_ is the dependent variable representing SES of households ‘i’ whereas *X*_*i*_, *Z*_*i*_ and *N*_*i*_ are the independent regressors in our model. *X*_*i*_ which is divided into the four main sources of income in village ‘i’, *Z*_*i*_ represents the two main industries in village ‘i’, and *N*_*i*_ denotes the two impact variables of Covid-19 on households in village ‘i’. All the independent variables are chosen based on the socio-economic features of the villages and in accordance with the national statistics of China. The variables are further explained in Eq 4. Moreover, *α*_0_ is the constant in the model while *β*_*i*_, *γ*_*i*_ and *δ*_*i*_ are the relevant coefficient measures. *μ*_*i*_ is the overall error term in the model.

To further understand the impacts of COVID-19 on the counties and the resilience of the poor household against the outbreak, we add additional variables and modify the model. The new variables reduce the bias and increase the explanation effect of the regressors in the model. We fit our final model as follows:

$$Logit\;SESi=\alpha_{0}+\beta_{1}{yfarm}_{i}+\beta_{2}{ybusn}_{i}+\beta_{3}{yemp}_{i}+\beta_{4}{ygov}_{i}+\gamma_{1}{indagri}_{i}+\gamma_{2}{indlivst}_{i}+\delta_{1}{sagri}_{i}+\delta_{2}{clivestk}_{i}+\varepsilon_{i}{res}_{i}+\rho_{i}{pov}_{i}+\tau_{i}{policyloan}_{i}$$ (4)

Where,

*yfarm*_*i*_: Income from farmland in the household ‘i’

*ybusn*_*i*_: Income from doing business in household ‘i’

*yemp*_*i*_: Income from local employment in household ‘i’

*ygov*_*i*_: Income from Government Transfer payments in the household ‘i’

*indagri*_*i*_: Household member associated with the agricultural industry

*indlivst*_*i*_: Household member associated with the livestock industry

*sagri*_*i*_: Supply of agricultural products during the pandemic

*clivestk*_*i*_: Cost of livestock feed during the pandemic

*res*_*i*_: Resilience of household ‘i’ against COVID-19

*pov*_*i*_: Ability of household ‘i’ to counter falling to poverty during the pandemic

*policyloan*_*i*_: Desired policy of loans for business on lower interest rate by household ‘i’

The new variable *res*_*i*_ represents the resilience of the household ‘i’ against COVID-19, *Pov*_*i*_ shows the susceptibility of the household ‘i’ falling into poverty and *policyloan*_*i*_ denotes the desired policy measures by the households in county ‘i’. *ε*_*i*_, *ρ*_*i*_ and *τ*_*i*_ are the respective coefficients measures of the newly added regressors.

We estimated our model using step-wise binary logistic regression. As our independent variable is binary coded and independent variables are categorical, it is an appropriate method for estimation. The model was also tested for reliability and validity through tests for consistency and goodness of fit for the factors. The results show significant estimates and in line with our assumptions and research questions. The outcomes of the analysis are reported in the next part.

## Results and findings

### Sample characteristics and descriptive summary

In our sample, most respondents belong to high SES status level (74%). In terms of per capital annual disposable income during normal years, most participants reported less than RMB 13000 (61%) while only 12% of participants had income higher than RMB 20000. Further, in terms of main income sources, most survey participants were dependent on government transfer payment (89%), followed by business (66%), local employment or wage income (41%), and family farming (25%). Additionally, 59% reported that they are mainly involved in agricultural industry. When asked about supply of agricultural materials (in comparison to last year), 61% respondents reported that they are receiving adequate supply of materials. However, only 1% pointed out that there is severe shortage.

Additionally, 67% of participants said that due to COVID-19 outbreak, the cost of livestock breeding has increased. Similarly, when we asked survey participants to express the likelihood of the rural households to fall into poverty due to COVID-19 pandemic, 15% reported very high likelihood followed by high likelihood (46%). Likewise, when respondents were asked to express their resilience and ability to prevent returning to poverty independently without any external support, 46% reported that they possess strong resilience capability, while only 17% reported that they have no resilience capability.

Table 2 shows the statistical summary of the dependent and independent variables. We collected the data through an online questionnaire. A total of 555 households were observed and surveyed. Most of the variables including the dependent variable ‘SESi’, are binary coded with values of 0 or 1. Some variables including ‘hhres’, ‘fallpov’ and ‘sagri’ are ordinal or ranked assuming values of 0, 1, 2 or 3.

**Table 2:** Descriptive statistics.

	SES	yfarm	ybusn	yemp	ygov	Indagri	Indlivst	sagri	clivestk	polloan	hhres	fallpov
Mean	0.26	0.75	0.34	0.59	0.11	0.41	0.47	1.87	1.56	0.34	1.57	1.61
S. E. Mean	0.019	0.018	0.020	0.021	0.013	0.021	0.021	0.028	0.029	0.020	0.040	0.036
S.D.	0.437	0.436	0.474	0.493	0.315	0.492	0.500	0.649	0.680	0.472	0.933	0.843
Minimum	0	0	0	0	0	0	0	0	0	0	0	0
Maximum	1	1	1	1	1	1	1	3	2	1	3	2
Obs.	555	555	555	555	555	555	555	555	555	555	555	555

The mean value of the variables lies between 0 and 2 with minimum of 0.26 for the dependent variable ‘SES’. For ‘sagri’, the maximum mean value is 1.87. The standard errors of mean are low for all the variables and ‘ygov’ shows the lowest values of 0.013. The standard deviation for all the main variables is between 0 and 1. ‘hhres’ has the highest standard deviation of 0.933 followed by ‘fallpov’ 0.843. For the maximum and minimum values of the survey responses of the households, the codes range between 0 and 3. In case of the dependent variable, the code is in reverse order assuming 0 for positive response and 1 for negative response. The codes for independent variables present 0 for low level and 1, 2 or 3 for higher categories of responses. Overall, the data shows clear statistical values for the variables used in the estimation method.

**Fig 2:**
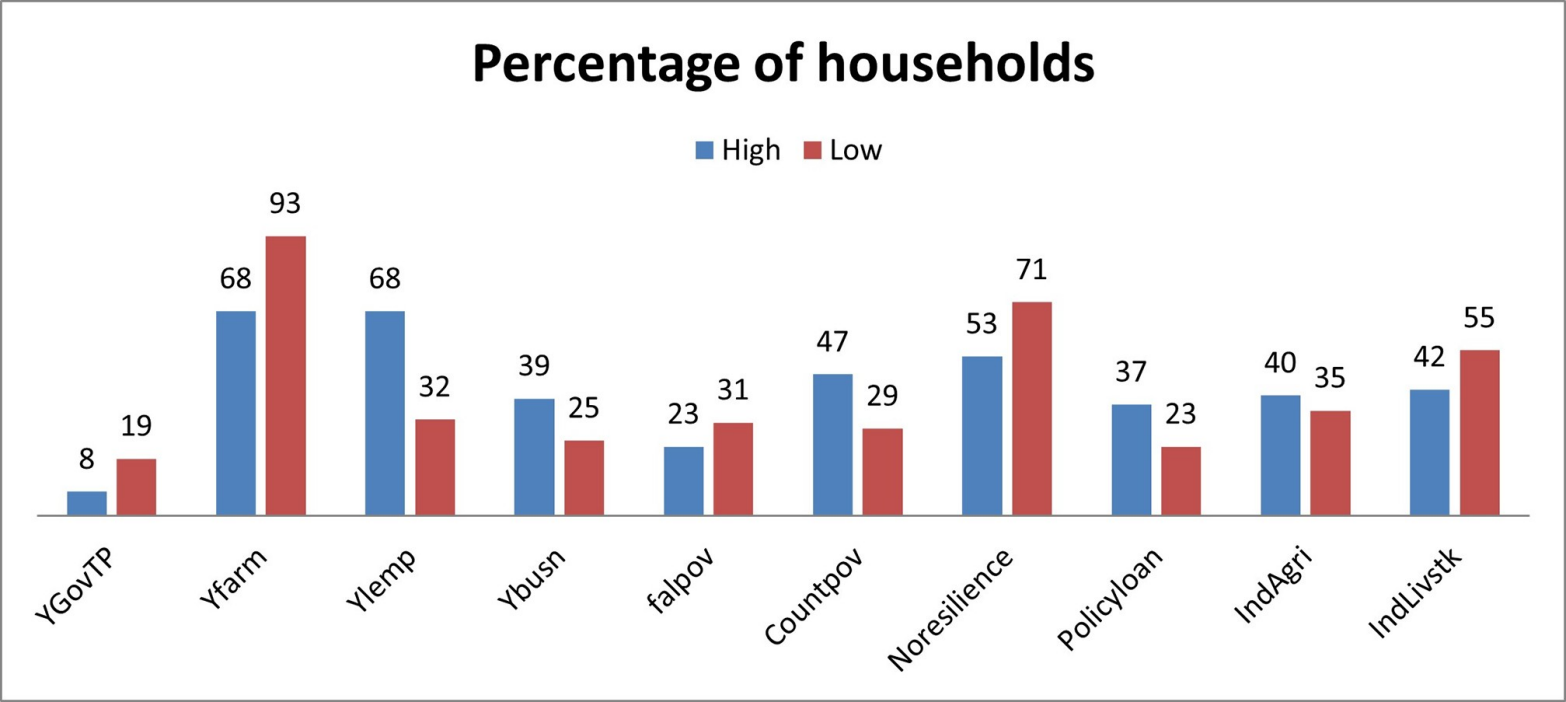
High and low SES households.

Fig 2 shows the difference in the SES of the households in percentages and the response of the households in case of socio-economic changes. Household with better socio-economic status rely on income from business (68% of high SES households) and local employment (39% high SES households) while households having low SES are more dependent on transfer payments from the government (19% of low SES households) and farm output (93% of low SES households).

Similarly, low SES households are more vulnerable (71%) and external shocks can drive them further into poverty (31%). In contrast, households with higher SES are more resilient (47%) and can cope with external shocks like COVID-19 regardless of external support. It also shows 35% and 55% of low SES households are engaged in agricultural and livestock industries respectively.

### Reliability and validity statistics

#### Cronbach’s alpha

The value of Cronbach’s alpha is calculated for the reliability or internal consistency of the estimations or scale. Cronbach’s alpha was first introduced by Lee Cronbach in 1951 in order to check the internal consistency of test [65]. In order for the results to be reliable and acceptable, the threshold value of Cronbach’s alpha should be between 0.7 and 0.95 [66, 67]. In our case, Cronbach’s alpha values for our models are above the threshold value of 0.7. The value implies that the variables used are reliable and consistent internally. The table 3 contains the Cronbach’s alpha values for the factors in each of our models.

**Table 3:** Cronbach’s alpha test of reliability.

Model	Cronbach’s Alpha	No. of Items
1	0.703	4
2	0.706	6
3	0.707	8
4	0.707	9
5	0.712	10
6	0.713	10

For testing of convergent validity, we use Factor Analysis for the four main components of all our factors, including 4 income factors in the first component, 2 industry factors as the second component, 2 cost factors as the third component, and the fourth component consists of 3 factors that have diverse loadings. The test is undertaken to check if the factors load are acceptable and distinct or not for each component. Table 4 shows the loading of the main components.

**Table 4:** Component matrix.

Variable	Component			
	1	2	3	4
Yfarm	0.715	-0.184	-0.115	0.115
YLemp	0.815	-0.011	-0.142	0.129
YBusn	0.761	0.007	-0.138	-0.118
YGovtTP	0.534	-0.063	0.379	0.160
IndAgri	0.234	0.272	0.660	0.088
IndLivstk	0.158	0.217	0.767	0.018
Sagri	0.081	0.836	-0.183	0.138
Clivestk	0.042	0.816	-0.135	0.158
Resilience	-0.011	-0.333	-0.095	0.634
Fallpoverty	0.135	0.116	-0.115	-0.780
PolicyLoan	0.207	-0.129	0.305	-0.405

Extraction Method: Principal Component Analysis.

Table 4 clearly depicts that the factors load distinctly for the four groups and confirms the validity of convergence among the main factors in each of the groups. The average factors loading for all the groups are also estimated to be higher than 0.5 threshold level and most of the groups average above 0.7 which is a good determinant of validity and also suggests convergent validity.

#### Goodness of fit

After fitting the model, it is important to analyze the fitness of the data in the model. There are different methods of calculating the goodness of fit like Pearson’s Chi-Square [68] and Likelihood ratio test [59, 60], but based on our data set, Hosmer-Lemeshow test for goodness of fit is considered a good way of presenting the fitness of our data into our model [69]. In the case of the Hosmer and Lemeshow test, the significant value should be above 0.05 for selection of the model. A lower significance value suggests the rejection of the model [70]. The results of the test are reported in Table 5.

**Table 5:** Hosmer and Lemeshow test.

Step	Chi-square	df	Sig.
1	5.584	6	0.471
2	5.271	8	0.728
3	2.649	8	0.954
4	3.503	8	0.899
5	6.520	8	0.589
6	6.556	8	0.585

According to Hosmer [69], the value of significance has to be above the threshold of 0.05% in order to determine a good fit. The null hypothesis assumes that the data is a good fit for the model. In our case, Table 5 clearly shows that the data fits our model, and our model has the goodness of fit as the significance of Hosmer-Lemeshow test estimates are above 0.05, which means we are unable to reject the model and our data fits the model. In addition, for the overall significance of the model, the Omnibus test of the Model coefficients also proved that coefficients of the independent regressors used in the model are significant. Table 6 presents the Omnibus test outcomes of the final estimations of the model.

**Table 6:** Omnibus tests of model coefficients.

	Chi-square	Df	Sig.
Step	265.793	10	0.000
Block	265.793	10	0.000
Model	265.793	10	0.000

### Main findings and discussions

Table 7 shows the stepwise binary logistic regression trends for our model based on the survey of households in different villages of Sichuan Province.

**Table 7:** Binary logistic regression output.

**Variables**	**(1)**	**(2)**		**(3)**		**(4)**		**(5)**		**(6)**	
	B	B	Exp. (B)	B	Exp. (B)	B	Exp. (B)	B	Exp. (B)	B	Exp. (B)
**Yfarm**	1.035*** [2.816] (0.370)	0.989** (0.390)	2.688	0.975** (0.402)	2.650	1.004** (0.404)	2.730	1.087** (0.431)	2.965	1.117** (0.449)	3.056
**YLemp**	-1.236*** [0.291] (0.243)	-1.228*** (0.250)	0.293	-1.114*** (0.259)	0.328	-1.075*** (0.261)	0.341	-1.051*** (0.282)	0.349	-1.172*** (0.303)	0.310
**YBusn**	-0.888*** [0.411] (0.277)	-0.917*** (0.284)	0.400	-0.900*** (0.292)	0.406	-0.826*** (0.295)	0.438	-0.776** (0.322)	0.460	-0.820** (0.330)	0.441
**YGovtTP**	1.512*** [4.536] (0.336)	1.611*** (0.346)	5.007	1.613*** (0.350)	5.016	1.647*** (0.357)	5.191	1.590*** (0.403)	4.902	1.491*** (0.420)	4.440
**IndAgri**	-	-0.704*** (0.237)	0.494	-0.789*** (0.244)	0.454	-0.786*** (0.245)	0.456	-.618** (0.271)	0.539	-0.664** (0.291)	0.515
**IndLivstk**	-	0.690*** (0.234)	1.993	0.796*** (0.243)	2.216	0.799*** (0.243)	2.223	0.705*** (0.272)	2.024	0.745** (0.296)	2.107
**Sagri**	-	-	-	-0.609*** (0.178)	0.544	-0.633*** (0.180)	0.531	-0.697*** (0.195)	0.498	-0.958*** (0.220)	0.384
**Clivestk**	-	-	-	0.474*** (0.190)	1.607	0.448** (0.192)	1.566	0.567*** (0.212)	1.763	0.593** (0.236)	1.809
**PolicyLoan**	-	-	-	-	-	-0.548** (0.255)	0.578	-0.580** (0.279)	0.560	-	-
**Resilience**	-	-	-	-	-	-	-	-1.138*** (0.147)	0.321	-1.167*** (0.157)	0.311
**Fallpoverty**	-	-	-	-	-	-	-	-	-	-1.198*** (0.178)	0.302
**Constant**	-1.268*** (0.385)	-1.319*** (0.389)	-	-1.074** (0.571)	--	-0.895 (0.575)	-	0.455 (0.637)	-	2.540*** (0.766)	-
**Observations**	555	555	-	555	--	555	--	555	--	555	--
**Nagelkerke R Square**	0.240	0.275	-	0.320	--	0.330	--	0.471	--	0.560	-

**Note:** Standard errors in parentheses

*** *p*<0.01

** *p*<0.05

* *p*<0.1

As our dependent variable ‘SES’ assumes binary values (0, 1), therefore, binary logistic regression analysis is adopted for the estimation of the coefficients in our model. The binary logit regression provides better estimations and is recommended in case the dependent variable consists of binary values. The use of SES as the representation of the households proved to be a better indicator of showing the impacts of shock on different households’ and their response to an external shock (COVID-19) as it takes into account the main economic factors relevant to households. All the independent variables are regressed and estimated for the effects on the dependent variables.

The table also shows the coefficients ‘B’ and the exponential form of the coefficients ‘Exp (B)’ of all the independent variables in the model. Exp (B) interprets the odds of the effects of the independent variables in case of the binary dependent variable.

#### Income effect

The variables ‘Yfarm’, ‘Ylemp’, ‘YBusn’, and ‘YGovt’ depict different household income sources in the villages. The significant income effect on the households can be seen from the Table 7 as all the variables are significant. Yfarm and YGovt are negative in relationship while Ylemp and YGovt attain positive sign concerning the dependent variable. The negative sign explains the odds of the independent variables to be less likely being the poor household while positive sign denotes higher odds of the effects on poor households. The Exp(B) values 3.056 and 4.440 for Yfarm and YGovt respectively demonstrate the odds of income earnings from farmland is 3.056% more for poor households while odds of depending on transfer payments from the Government are about 4.44% higher in poor households as compared to households that are non-poor with high SES.

The difference indicated by these estimations clearly suggests that poor households are more vulnerable with subsistence source as farmland income and thus more dependent on support from the government for income and livelihood while non-poor household is less vulnerable due to better and continuous income sources like a business and local employment. The differences suggest that non-poor and higher SES households are characterized by income from business and employment. Business and Employment show a key and significant difference between the low and high SES households. Such means and sources of income are estimated to have a positive impact on the SES of the households.

#### Effect on prices and costs

The result provided the changes in prices and costs of agricultural products and the impacts of such changes on the different households. The significant results for agricultural industry (-0.704) and supply of agricultural products (-0.609) suggest that the odds of the poor household member working in the agricultural industry and the effect on supply of agricultural products are less likely compared to non-poor households. There is a clear distinction as working in an agricultural industry may provide higher income opportunities, and thus, may increase the SES. Similarly, the agricultural supply may be increase for non-poor households due to the shock as non-poor household demand for agricultural products may increase due to the fear of pandemic. These findings can be attributed to the fact that non-poor households depend on the supply of agricultural products, and thus, the effect on the supply of agricultural products may be higher in non-poor households. The possible explanation may be that China’s agricultural sector has been on the verge of modernization, which can be focused on future policy in giving opportunities for low-income families to be part of the agricultural industry. In addition, without the necessary source of income, the poor family may only end up decreasing marginal income with the higher opportunity cost of migration work in non-agricultural sector in town or else; while a non-poor family is in an opposite situation of its counterpart. Moreover, if the poor’s situation cannot be reversed in the long run, the rural population would be separated into the Agri-business group and the city worker group, which may be the natural selection process.

In contrast, household members working in the livestock industry and higher livestock cost are most likely to happen in the case of poor households. The finding also highlights that poor household members are about 2% more likely to be associated with livestock industry than non-poor households. It can be attributed to the fact that most of the poor households depend on livestock and products from livestock for daily consumption and livelihood. As a result of the shock, the dependence of poor households on livestock’s increase and thus the costs for feed rise.

#### Resilience and vulnerability on SES

Further, estimates for the resilience of community (-1.167) in tackling the pandemic explain that the odds of households to be resilient against pandemic are less likely to be in low SES households, which can be extended as households in non-poor households tend to be more resilient towards COVID-19 due to better sources of income. Similarly, the odds of countering falling into poverty are less in poor households as compared to non-poor households. Low SES households mostly depend on income from farms and support from the government. It is unlikely for the poor households to overcome any external shock or show resilience without the support of the government. Once such households are affected by an outbreak, then the chances of these households falling into poverty increases as these households depend on subsistence sources of income and government support and assistance.

#### Policy effect

In terms of the policy, the provision of loans on lower interest rates for business based on the coefficient value of -0.580 explains the odds of households desiring business loans on low-interest rates are less likely to in poor households. Non-poor households tend to establish business and earnings from employment, while households with low SES are less likely to be engaged in business and employment activities. These factors can enable households to earn higher income which in this case, the higher desire for loans for business purposes in non-poor households proves the difference between poor households and non-poor households.

The overall significance of the coefficients and the goodness of fit for variables in the model were explained previously. For explanation and predictive power of the independent variables, the binary logistic regression calculates Nagelkerke R Squared values. The value for Nagelkerke R Squared in our final model is 56%, which shows the independent variables in our model are good in specification and estimations.

For further analysis and checking of the model, we sort the data and estimate the variables in categorical forms. The categorical estimations of the model provide significant outcomes as compared to the regression analysis of the overall effects of the variables. The estimates of the coefficients in our model are presented in Table 8.

**Table 8:** Binary logistic regression (categorical variables).

Variables	-1		-2		-3	
	B	Exp(B)	B	Exp(B)	B	Exp(B)
**Yfarm(1)**	0.998**	2.712	1.108***	3.027	0.871*	2.39
	-0.39		-0.412		-0.518	
**YLemp(1)**	-1.201***	0.301	-1.067***	0.344	-1.543***	0.214
	-0.251		-0.264		-0.372	
**YBusn(1)**	-0.841***	0.431	-0.819***	0.441	-0.767**	0.465
	-0.286		-0.297		-0.385	
**YGovtTP(1)**	1.627***	5.088	1.639***	5.15	1.747***	5.74
	-0.352		-0.357		-0.532	
**IndAgri(1)**	0.705***	2.024	0.798***	2.222	0.525	1.691
	-0.238		-0.249		-0.35	
**IndLivstk(1)**	0.697***	2.009	0.799***	2.222	0.682**	1.978
	-0.235		-0.246		-0.347	
**PolicyLoan(1)**	-0.539**	0.584	-0.583**	0.558	-0.741**	0.477
	-0.249		-0.259		-0.373	
**Sagri**	-	-	***	-	***	-
**Sagri(1)**	-	-	1.313	3.715	1.13	3.097
			-0.807		-1.181	
**Sagri (2)**	-	-	0.851**	2.343	1.352**	3.864
			-0.397		-0.583	
**Sagri (3)**	-	-	-0.248	0.78	-0.296	0.744
			-0.361		-0.538	
**CLivestk**	-	-	0.460**	1.584	0.448	1.565
			-0.194		-0.284	
**Resilience**	-	-	-	-	***	-
**Resilience(1)**	-	-	-	-	1.941***	6.965
					-0.412	
**Resilience(2)**	-	-	-	-	-1.794***	0.166
					-0.416	
**Resilience(3)**	-	-	-	-	-3.997***	0.018
					-1.114	
**Fallpoverty**	-	-	-	-	***	-
**Fallpoverty(1)**	-	-	-	-	-0.786	0.455
					(0.530	
**Fallpoverty(2)**	-	-	-	-	-2.115***	0.121
					-0.544	
**Fallpoverty(3)**	-	-	-	-	-3.905***	0.02
					-0.812	
**Constant**	-1.910***	-	3.054***	0.047	-0.735	0.48
	-0.457		-0.65		-0.942	
**Observations**	555	-	555	-	555	-
**Nagelkerke R Square**	0.286	-	0.345	-	0.701	-
**Pearson Correlation**	-	-	-	-	0.61	-

Standard errors in parentheses

*** *p*<0.01

** *p*<0.05

* *p*<0.1

The estimations in the table show different categorical estimates for our main variables in the model. The results are similar to the non-categorical estimations with a few changes suggesting robust outcomes in our main model. The income effect and industrial effects of the variables show similar signs as the previous model. Similarly, the Nagelkerke R Square value is also high at 70.1%, which shows a higher predictive power of the variables. As in the case of Binary logistic regression, the Nagelkerke R Square values loosely define the effect of independent variables, therefore, we also calculated the correlation value of the probabilities of our independent variables and the dependent variable using the Pearson Correlation test. The value of the Pearson Correlation test is 61%, which is also high and shows a good choice of our independent variables in explaining the dependent variable.

The resilience has an overall significant impact in determining the odds of non-poor households to be more resilient as compared to poor households. The first category representing resilience in the table suggests that there is no resilience. Based on the estimation value of 1.941 it can be interpreted, as the odds of households with no resilience are about 1.9 times more likely to be poor and low SES households. Based on exp(B) value of 6.965 it can also be explained that a household with no resilience is approx. 6.96% more likely to be poor, which is highly significant. On the contrary, the resilience coefficients for 2^nd^ and 3^rd^ category we obtain the odds ratios of 0.166 and 0.018. Thus, the odds of a household to be resilient or highly resilient are less likely to be poor households.

Similar trends are represented in terms of countering poverty. The signs are negative showing less likeliness towards poor households, and exponential coefficients for the three categories assume values of 0.455, 0.121, and 0.020, respectively. All the category estimations were negative, predicting less likeliness of being poor or low SES households. The estimates were significant except for the first category. The results suggested that the odds of households countering poverty strongly or very strongly are less likely to be a poor household. It can be explained that a household with low SES is more vulnerable to the impacts of COVID-19 and may fall back below the poverty line, while the high SES households show more resilience and less vulnerability towards the pandemic. The results further highlight that the number of new poor may increase in the future if the pandemic continues as these households are less resilient and less likely to counter falling back into poverty.

**Fig 3:**
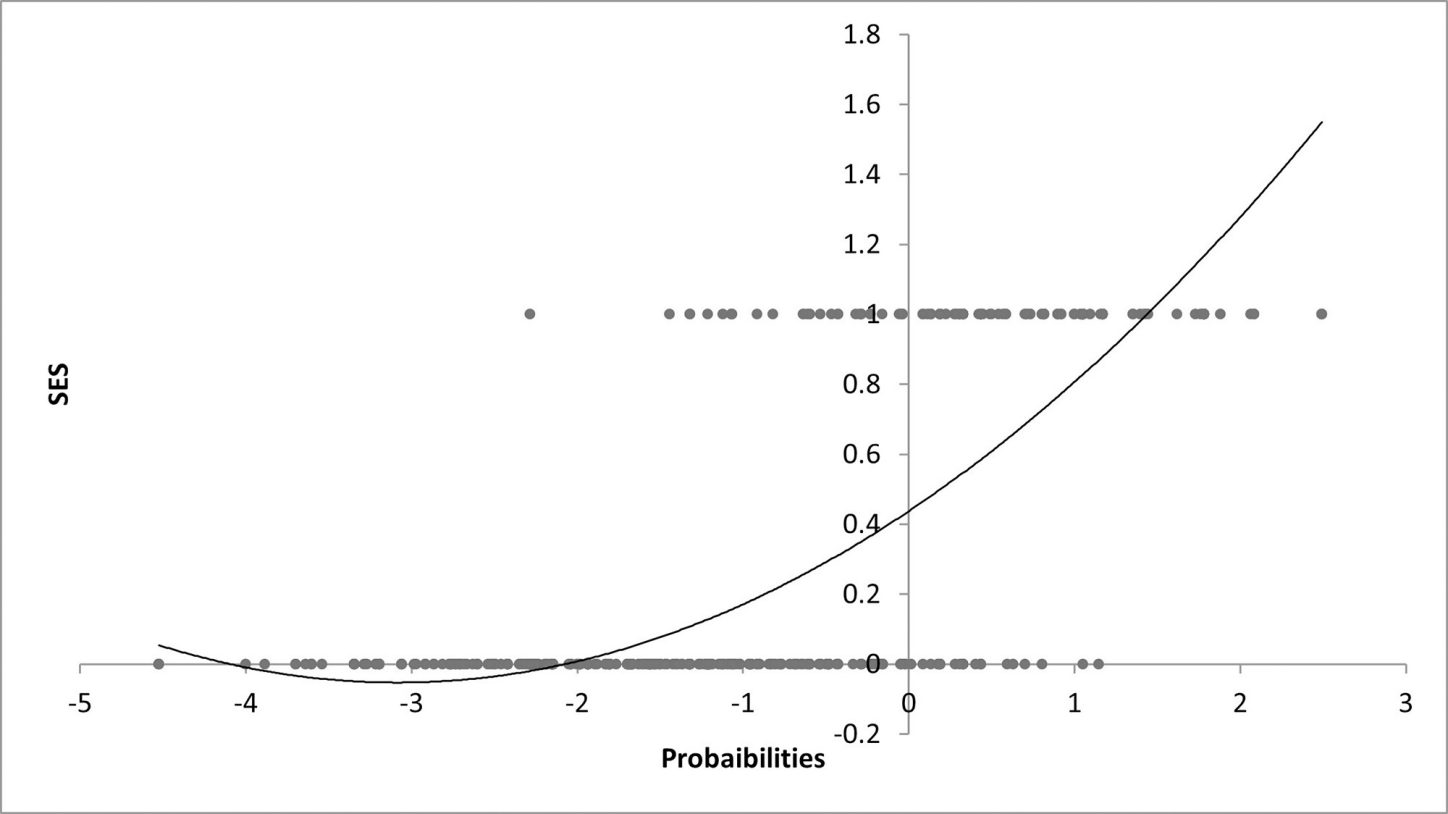
Predicted probabilities and SES.

**Fig 4:**
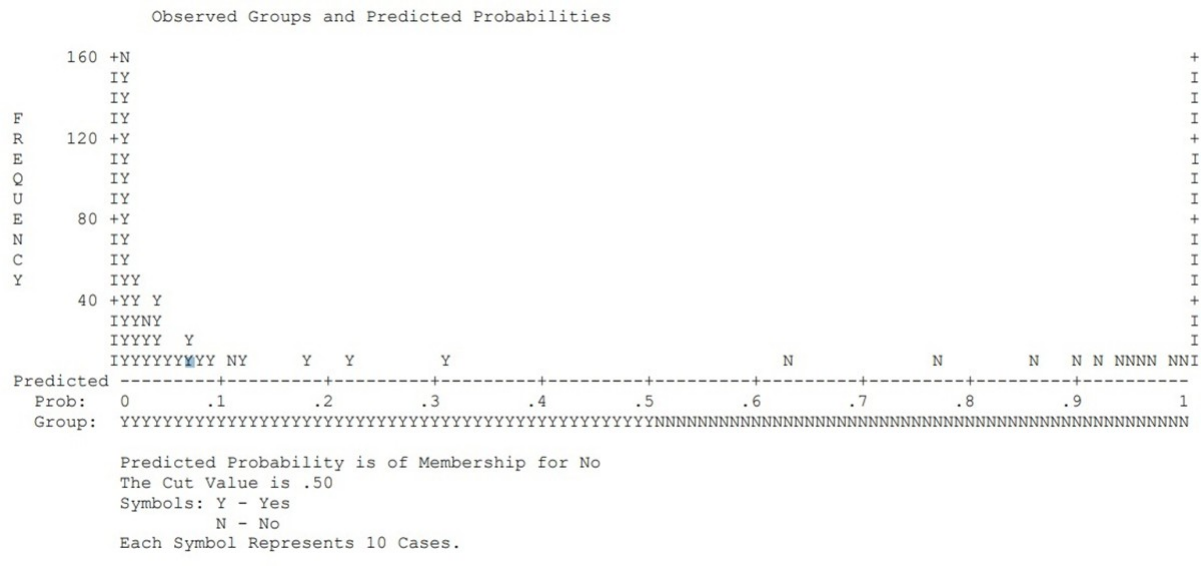
Predicted probabilities. Piling of the data responses and predicted probabilities on both sides of the figure explains a good outcome related to the variables and the model.

For further explanation of the model, we estimated the predicted probabilities of the variables against the dependent variable. Fig 3 shows the predicted probabilities of the independent variables against the values of dependent variables. Although some values overlap, the majority of the distribution provides a good prediction of the model. It predicts that households with low SES are likely to fall on the positive side while a household with high SES over the negative side. The same outcomes are also presented in Fig 4.

## Discussion

This paper examined the difference in impacts of COVID-19 on households based on the socio-economic differences in the villages of Sichuan province, China. Our main goal was to identify the economic factors influencing households’ responses to COVID-19 based on socio-economic differences. The SES provides a better and deeper knowledge of the changes in household economic conditions and indicators as a result of an outbreak or health crisis like COVID-19. This study also assesses the likelihood of the households’ SES conditions to see if there are any disparities in the level of resilience to COVID-19 and households’ ability to avoid falling into absolute poverty.

The outcomes presented that households are less likely to be affected or the impact of COVID-19 on non-poor households is lower as compared to households that are poor with lower SES. Similarly, Ndirangu [6] argued that in the case of developing economies, people living in low-income households face dual misfortunes: income-generating difficulties and vulnerability to socio-economics crises. Likewise, Jalan & Ravallion [7] also argues that households with higher SES have greater access and a broader asset base to insurance and credit services, which will help cushion them against the effects of an external shock, like COVID-19. The chances of falling back into the poverty of a poor household are significantly higher than the Non-poor household, which suggests that the numbers of new poor as mentioned in recent articles are estimated to increase more due to the pandemic [71]. Further, Adler & Snibble [11] argue that in comparison to households with lower SES, higher SES households have better access to health care, housing, knowledge, and nutrition.

In the case of resilience, response, and tackling the economic influence of COVID-19, the estimate predicts that the odds of non-poor households (high SES) to be resilient against COVID-19 are higher in contrast to low SES households. Business and employment may be better sources of income, and industry plays key roles in determining the SES of the households, which can affect the resilience of the communities towards recovery during the pandemic.

## Implications

Based on the outcomes of the paper and literature studies, it is observed that poor households and people having dependent on farm income are affected more and are more dependent on external support; therefore, it is necessary to devise policies for supporting and creating new jobs and income generating opportunities for them. Focusing on agricultural sector modernization and providing opportunities in the agricultural industry may contribute both at the household and national level. Small scales agricultural industries can be set up according to the needs of the community or county are expected to be of higher significance. Loans on lower interest rates for businesses can increase resilience and decrease the vulnerability of the poor households with access to funds. These policies can have better and long-lasting impacts on changing the livelihoods and SES level of the poor communities. At a general level, the government bodies and institutions provide relief and support to the poor households and vulnerable communities with low income and low employability [2]. Such policies and support should be temporary, as households depending on government support have a higher chance of falling or staying in the low SES category and may not divulge into other income opportunities.

## Limitations

Despite insightful implications, this research is not without limitations. Primarily, this study was cross-sectional in nature which does not allow to make causal inferences. To make causal inferences, longitudinal studies are needed. Furthermore, the sample was obtained through the snowball sampling technique, which provides room for further research. Concerning limitations during the course of the research, there are some areas that may be improved in future research articles. The sample we collected was based on a single province due to time restraints; other researchers can increase the data set and collect a larger sample, a country-level data, which will give outcomes that are more robust. Similarly, since the lockdown policies and pandemic are ongoing; more research and post-pandemic scenario will provide better research opportunities and unbiased estimates for future researchers.

## Supporting information

S1 File (DOCX)
