# Supplementary material for: Socio-economic status, resilience, and vulnerability of households under COVID-19: Case of village-level data in Sichuan province
Source: PLoS One. 2021 Apr 29;16(4):e0249270. doi: 10.1371/journal.pone.0249270 (PMC8084142; doi:10.1371/journal.pone.0249270)
Supplement: S1 File — (DOCX) [file pone.0249270.s001.docx]

S1 File.

**(Study Questionnaire)**

SOCIO-ECONOMIC STATUS, RESILIENCE, AND VULNERABILITY OF HOUSEHOLDS UNDER COVID-19: CASE OF VILLAGE-LEVEL DATA IN SICHUAN PROVINCE

**Respondent No.** _______ **Date:** ______________

The questionnaire is developed to study and investigate the economic impacts of COVID-19 on households based on differences in the socio-economic status (SES). In this study we attempt to determine the household-level effects of the COVID-19 shock using different socio-economic factors. For this purpose, we are using this survey questionnaire to collect relevant data from different households in villages mostly located in Sichuan Province, China.

**Note:** Please fill the form and choose the appropriate responses to question 2 – question 12.

1. **The village/community belongs:**

Province: ______________________________

City/State: ______________________________

County/Village: ______________________________

1. **What is the socio-economic status of the household in terms of national standards?**
2. High ii) Low
3. **Whether the household is a poor household?**
4. Yes ii) No
5. **There are _____ villager groups / community groups in the village community (County)**
6. 3-5 ii) 6-7 iii) 8-10 iv) More than 10
7. **The per capita annual disposable income of the households in normal years is probably _____**
8. < 10000 ii) 10001-13000 iii) 13001-15000 iv) 15001-20000 v) 20001-25000
9. **The main source of income for the households in the village/community**
10. Family Farming Income
11. Yes b) No
12. Income from operating a business locally or abroad

a) Yes b) No

1. Local employment income or wage income

a) Yes b) No

1. Government Transfer Payment

a) Yes b) No

1. **What are the main industries to which members of the households in the village / community belong to_______**
2. Agricultural products industry (vegetables, fruits)
3. Livestock industry (breeding pigs, chickens, ducks, etc.)
4. **Compared with the same period last year, currently in the stage of spring cultivation, what is the level of supply of agricultural materials as a whole ____**
5. Severe Shortage
6. Slight shortage
7. Relatively adequate
8. Abundant
9. **After the outbreak, the cost of livestock breeding has _____**
10. Declined ii) Remained the same iii) Increased
11. **Do you think that this new corona pneumonia epidemic will cause the rural households to fall into poverty _____**
12. Yes ii) May be iii) NO
13. **The level of ability that the rural household possess to show resilience and to prevent returning to poverty independently without external support**
14. No ii) Weak iii) Strong iv) Very Strong
15. **Do you think the policy of providing loans on lower interest rates is an effective way to support the household in order to prevent it from returning to poverty after the pandemic?**
16. Yes ii) No

**Thank you for your valuable feedback and time!**
